# Diversity and distribution patterns of non-volant small mammals along different elevation gradients on Mt. Kenya, Kenya

**DOI:** 10.24272/j.issn.2095-8137.2019.004

**Published:** 2018-12-14

**Authors:** Simon Musila, Zhong-Zheng Chen, Quan Li, Richard Yego, Bin Zhang, Kenneth Onditi, Immaculate Muthoni, Shui-Wang He, Samson Omondi, James Mathenge, Esther N. Kioko, Xue-Long Jiang

**Affiliations:** 1 Mammalogy Section, Department of Zoology, National Museums of Kenya, Nairobi 40658-00100, Kenya; 2 Kunming Institute of Zoology, Chinese Academy of Sciences, Kunming Yunnan 650223, China; 3 Anhui Province Key Laboratory for Conservation and Exploitation of Biological Resource, College of Life Science, Anhui Normal University, Wuhu Anhui 241000, China; 4 Kenya Wildlife Service, Mweiga Research Station, Nyeri 753-10100, Kenya; 5 Sino-Africa Joint Research Center, Chinese Academy of Sciences, Nairobi 62000-00200, Kenya

**Keywords:** Small mammals, Species richness, Abundance, Elevation, Mt. Kenya

## Abstract

The distribution of small mammals in mountainous environments across different elevations can provide important information on the effects of climate change on the dispersal of species. However, few studies conducted on Afromontane ecosystems have compared the altitudinal patterns of small mammal diversity. We investigated the species diversity and abundance of non-volant small mammals (hereafter ‘small mammals’) on Mt. Kenya, the second tallest mountain in Africa, using a standard sampling scheme. Nine sampling transects were established at intervals of 200 m on the eastern (Chogoria) and western (Sirimon) slopes. A total of 1 905 individuals representing 25 species of small mammals were trapped after 12 240 trap-nights. Abundance was highest at mid-elevations on both slopes. However, species richness and their distribution patterns differed between the two slopes. More species were recorded on Chogoria (24) than on Sirimon (17). On Chogoria, species richness was higher at mid-high elevations, with a peak at mid-elevation (2 800 m a.s.l.), whereas species richness showed little variation on the Sirimon slope. These results indicate that patterns of species diversity can differ between slopes on the same mountain. In addition, we extensively reviewed literature on Mt. Kenya’s mammals and compiled a comprehensive checklist of 76 mammalian species. However, additional research is required to improve our understanding of small mammal diversity in mountain habitats in Africa.

## INTRODUCTION

Baseline information on biodiversity is important in ecosystem management planning. Clarifying species distribution provides useful information on ecology, habitat preference, and species replacement (Goodman et al., 1996; Malonza et al., 2018; Stanley & Kihaule, 2016). Studies investigating the relationship between species richness and altitude have yielded diverse patterns (Taylor et al., 2015). For instance, previous studies on non-volant small mammals have observed hump-shaped diversity curves, with the highest richness found at mid-elevations (McCain, 2005). The montane mammal communities of sub-Saharan Africa have been sporadically studied, with research on mountain elevation distribution relatively well documented, including that of the Ruwenzori Mountains (Kerbis et al., 1998), Udzungwa Mountains (Stanley & Hutterer, 2007), and Mt. Kilimanjaro (Mulungu et al., 2008; Stanley et al., 2014). However, studies of small mammals along different elevation gradients on Mt. Kenya are lacking.

Mt. Kenya is the second tallest mountain in Africa and one of the most important ecosystems for the conservation of biodiversity. It is recognized internationally both as an Important Bird Area and World Heritage site and is a vital water catchment area in Kenya (Bennun & Njoroge, 1999). The mountain has diverse habitats, including montane forest, bamboo, moorland, and alpine tussock grasslands (Happold & Happold, 1989; Yalden, 1988; Young & Evans, 1993). Although several notable studies have been conducted on the diversity of mammals on Mt. Kenya (Coe & Foster, 1972; Hollister, 1918, 1919; Moreau, 1944; Thomas, 1900; Young & Evans, 1993), these previous surveys were of short duration or opportunistic and did not focus on small mammal elevational distribution (Coe & Foster, 1972). Information on the distribution of animals along different elevations on Mt. Kenya can be used as a baseline for monitoring future changes in the distribution of species, especially due to the effects of climate change, compared with that in previous collections (e.g., Hollister, 1918, 1919). In the present study, we conducted a comparative analysis of small mammal species richness on the Chogoria and Sirimon slopes of Mt. Kenya to understand their diversity and distribution patterns.

## MATERIALS AND METHODS

### Study area

**Figure 1:**
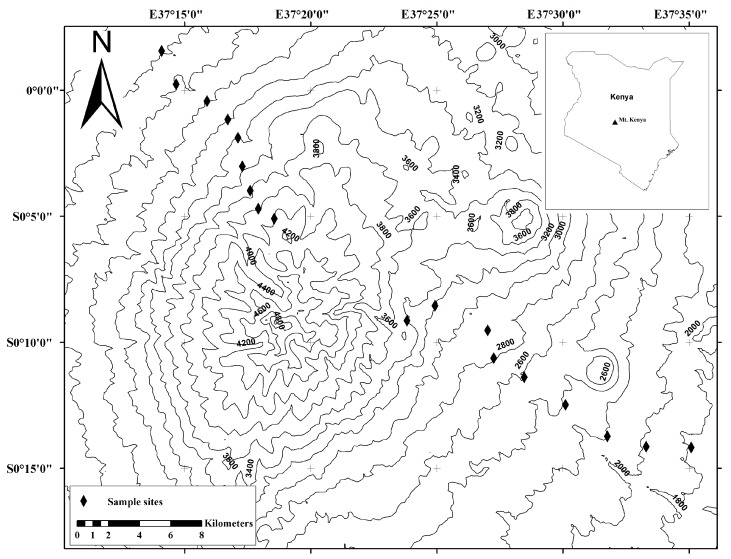
Map of Mt. Kenya showing the nine elevation transects sampled on the Chogoria and Sirimon slopes

This study was conducted on Mt. Kenya in September and October 2015. The mountain is located at S0°10′, E37°20′ and has an altitudinal range of 1 600–5 200 m a.s.l. (Bennun & Njoroge, 1999). The Kenya Forest Service manages the lower elevation forests, whereas the moorland (above 3 000 m a.s.l.) is managed as a National Park (71 500 ha) by the Kenya Wildlife Service (Bennun & Njoroge, 1999). The forest has an estimated total area of 271 000 ha. Blackett (1994) estimated that of the 199 500 ha of forest reserve area, closed canopy forest accounted for 61 000 ha, bamboo forest mosaic for 63 000 ha, forest scrub for 20 000 ha, exotic plantation forest for 20 500 ha, and non-forest habitat for 35 000 ha. The windward eastern slope of the mountain includes much of Chogoria and Meru towns, whereas Nanyuki and Naromoru towns are found on the leeward western slope. The current study was undertaken on both slopes. Highland forest starts at 1 800 m a.s.l. on Chogoria, but at 2 400 m a.s.l on Sirimon (Figure 1).

### Sampling methods

We conducted small mammal surveys from 1 800 m to 3 400 m a.s.l. on the Chogoria slope and from 2 400 m to 4 000 m a.s.l. on the Sirimon slope (Figure 1). The broad vegetation characteristics of each elevation gradient are briefly described in Table 1. Sampling stations were established at nine sites at intervals of 200 m on both slopes (Figure 1, Table 1). At each site, six trap lines were established, each with 25–30 trap stations at intervals of 10 m. At each trap station, one Sherman trap and one metallic snap trap was set at 1–2 m apart. The Sherman traps were baited with oat flakes, whereas the snap traps were baited with raw peanuts. In addition, six bucket pitfalls were used to capture shrews along each trap line. Traps were set in microhabitats with suspected small mammal occurrence, including under tree logs, along rodent runs, in thick grass, or under shaded vegetation. Traps were checked once in the morning, re-baited and left in their original position for two consecutive nights, before being moved to the next transect. Six hundred and eighty (680) trap nights were accumulated at each transect. Measurements were taken for each individual, including lengths of head and body (HB), tail (TL), hind foot (HF), and left ear (EL), as well as weight (WT). Vouchers of each species were prepared either as stuffed museum specimens or preserved whole as wet specimens in 70% ethanol and deposited in the Mammalogy Section Laboratory, National Museums of Kenya (NMK). For each specimen, muscle and/or liver tissue samples were obtained and preserved in absolute ethanol for further DNA work. We also recorded large- and medium-sized mammals encountered during the survey by direct (sightings) or indirect observation (scats and footprints). In addition, we extensively reviewed literature on Mt. Kenya’s mammals to compile a list of all mammals (Baker, 1971; Bennun & Njoroge, 1999; Butynski & de Jong, 2007; Carleton & Byrne, 2006; Coe & Foster, 1972; Flux & Flux, 1983; Hollister, 1918, 1919; Kingdon et al., 2013; Mbau et al., 2009, 2010; Monadjem et al., 2015; Musila et al., 2019; Musser & Carleton, 2005; Stanley et al., 2015; Taylor et al., 2011; Young & Evans, 1993). Taxonomic statuses and species names followed Kingdon et al. (2013) and Musila et al. (2019).

**Table 1:** Broad vegetation characteristics of the sampled elevation gradients on the Chogoria and Sirimon slopes of Mt. Kenya

Slope	Altitude (m a.s.l.)	Broad vegetation types
Transects along Chogoria slope		
1	1 800	Mixed plantation – indigenous trees, and exotic (*Eucalyptus, Grevillea*) trees
2–4	2 000, 2 200 and 2 400	Mixed indigenous forest
5	2 600	Bamboo habitat with canopy dominated by very few *Podocarpus latifolius* and understory covered mainly by mature (5–10 m high) Bamboo *Arundinaria alpine*. Some sections of bamboo were regenerating with young bamboo after it was destroyed by fire in 2013
6	2 800	Bamboo habitat with canopy dominated by very few *Podocarpus latifolius* and understory covered mainly by mature (5–10 m high) Bamboo *Arundinaria alpine*. Some sections of bamboo were regenerating with young bamboo after it was destroyed by fire in 2013
7	3 000	*Juniperus*–*Hagenia* habitat with canopy dominated by *Hagenia abyssinica* and large patches of tussock grasslands
8	3 200	Moorland habitat dominated by *Erica* and *Stoebe*, as well as large patches of tussock grasslands
9	3 400	Moorland habitat dominated by *Adenocarpus*, *Protea*, *Helichrysum* and *Erica* bushes, as well as large patches of tussock grasslands.
Transects along Sirimon slope		
1–2	2 400 and 2 600	Mixed trees indigenous forest
3	2 800	Mixed trees-bamboo forest
4	3 000	*Hagenia-*mixed trees forest
5	3 200	*Erica, Stoebe*–*Hagenia* habitat and some patches of tussock grassland
6–7	3 400 and 3 600	Moorland habitat dominated by *Erica*– *Dendrosenescio*– *Lobelia* with many tussock grasslands
8	3 800	*Dendrosenescio* moorland with many tussock grasslands
9	4 000	*Dendrosenescio* plants of alpine with many tussock grasslands

### Data analysis

All trapped individuals were identified to genus or species level based on external and craniodental morphology and distribution using Hollister (1919), Kingdon et al. (2013), and Monadjem et al. (2015). In addition, specimens that could not be identified to species level were further analyzed using molecular and morphometric data. We amplified the complete mitochondrial cytochrome *b* gene (cyt *b*) and/or cytochrome oxidase I gene (*COI*) for all specimens. We identified the haplotypes from both genes using DnaSP v.5.10 (Librado & Rozas, 2009). Phylogenetic relationships among the haplotypes were constructed by Bayesian inference in BEAST v1.8.2 (Drummond et al., 2012). Each major monophyletic clade in the Bayesian tree was recognized as a putative species. These putative species were then identified following Hollister (1918, 1919), Kingdon et al. (2013), and Monadjem et al. (2015), and by comparing the molecular and morphometric data with specimens in the NMK and Field Museum of Natural History collections. The number of individuals of each small mammal species was compiled for each transect and slope. The Shannon-Wiener index (Shannon et al., 1963) of diversity was used to calculate species diversity. All statistical analyses were performed using SPSS (IBM Corporation, 2013). Species identified from opportunistic surveys and literature review were not included in the analyses of species richness and distribution.

## RESULTS

### Small mammal species richness and distribution patterns

A total of 76 species of mammals were recorded from Mt. Kenya, including 35 from systematic and opportunistic surveys in the current study and 41 from the literature review. Of the 76 species, 46 were small mammals (less than 1.5 kg) and the rest (30) were medium- or large-sized mammals (Supplementary Table S1).

In the current study, we captured 25 small mammal species from three orders (Table 2). Many species (16) were shared between the two slopes, but more species were recorded on Chogoria (24) than on Sirimon (17). The mean number of species at each elevation was lower on Chogoria (9.00±2.50) than that on Sirimon (9.22±1.30). Although the number of species captured per elevation was more varied for Chogoria (4–12) than for Sirimon (7–11) (Table 3), the Shannon-Weiner index of diversity was lower for Chogoria (2.26±0.24) than for Sirimon (2.34±0.27).

**Table 2:** Species of small mammals captured on the Chogoria and Sirimon slopes of Mt. Kenya

No.	Order	Family	Species	Sirimon	Chogoria
1	Hyracoidea	Procaviidae	*Dendrohyrax arboreus*		✓
2	Rodentia	Sciuridae	*Paraxerus ochraceus*		✓
3	Rodentia	Gliridae	*Graphiurus murinus*	✓	✓
4	Rodentia	Gliridae	*Graphiurus* sp.^1^	✓	✓
5	Rodentia	Spalacidae	*Tachyoryctes splendens*		✓
6	Rodentia	Nesomyidae	*Dendromus insignis*	✓	✓
7	Rodentia	Muridae	*Lophuromys zena*	✓	✓
8	Rodentia	Muridae	*Dasymys incomtus*^2^	✓	✓
9	Rodentia	Muridae	*Grammomys gigas*	✓	✓
10	Rodentia	Muridae	*Hylomyscus endorobae*	✓	✓
11	Rodentia	Muridae	*Lemniscomys striatus*	✓	
12	Rodentia	Muridae	*Mus* sp.*		✓
13	Rodentia	Muridae	*Mus triton*	✓	✓
14	Rodentia	Muridae	*Oenomys hypoxanthus*		✓
15	Rodentia	Muridae	*Otomys orestes*	✓	✓
16	Rodentia	Muridae	*Otomys tropicalis*	✓	✓
17	Rodentia	Muridae	*Praomys jacksoni*	✓	✓
18	Rodentia	Muridae	*Rhabdomys dilectus*	✓	✓
19	Soricomorpha	Soricidae	*Crocidura allex*		✓
20	Soricomorpha	Soricidae	*Crocidura elgonius*^3^	✓	✓
21	Soricomorpha	Soricidae	*Crocidura montis*^4^	✓	✓
22	Soricomorpha	Soricidae	*Crocidura nigrofusca*^5^		✓
23	Soricomorpha	Soricidae	*Surdisorex polulus*	✓	✓
24	Soricomorpha	Soricidae	*Crocidura olivieri*		✓
25	Soricomorpha	Soricidae	*Sylvisorex mundus*	✓	✓
	**Total**			**17**	**25**

* Possible new species; ^1^ Could be *Graphiurus microtis saturatus* in Hollister, 1919; ^2^ Could be *Dasymys helukus savannus* in Hollister, 1919; ^3^ Could be *Crocidura allex alpina* in Hollister, 1918; ^4^ Could be *Crocidura fumosa fumosa* in Hollister, 1918; ^5^ Could be *Crocidura turba zaodon* in Hollister, 1918.

**Table 3:** Number of individuals and species richness at each elevational site on Mt. Kenya

Slope	Elevation (m a.s.l.)	Individual	Species richness	Shannon-Weiner indices
Chogoria	1 800	87	7	1.98
Chogoria	2 000	91	7	2.19
Chogoria	2 200	61	4	1.82
Chogoria	2 400	86	10	2.20
Chogoria	2 600	76	11	2.49
Chogoria	2 800	149	12	2.36
Chogoria	3 000	162	10	2.25
Chogoria	3 200	98	10	2.46
Chogoria	3 400	88	10	2.60
Sirimon	2 400	82	12	2.66
Sirimon	2 600	106	9	2.25
Sirimon	2 800	134	8	1.77
Sirimon	3 000	120	7	2.28
Sirimon	3 200	142	11	2.31
Sirimon	3 400	75	9	2.59
Sirimon	3 600	141	11	2.53
Sirimon	3 800	129	9	2.46
Sirimon	4 000	78	7	2.22
Total		1 905	25	

Elevational distribution varied among species and between slopes. Species in *Lophuromys* and *Crocidura* were the most abundant and were recorded at all sampling elevations on both the Sirimon and Chogoria slopes. One species in the genus *Mus* (hereafter ‘*Mus* sp.’) differed from any known form and may represent an undescribed species. The Mt. Kenya mole shrew (*Surdisorex polulus*), endemic to Mt. Kenya, was recorded from 2 400 to 3 400 m a.s.l., except for 2 600 and 3 200 m a.s.l., on Chogoria but at 3 200–3 800 m a.s.l. on Sirimon. *Lemniscomys striatus* was recorded at 2 400 m a.s.l. on Sirimon but not on Chogoria. *Dendromus insignis* was recorded from all elevation transects on Sirimon, except at 2 800 m a.s.l., but only at 3 000–3 400 m a.s.l. on Chogoria. *Praomys jacksoni* was recorded from three elevations between 2 400–2 800 m a.s.l. on Sirimon but in five sampling elevations between 1 800–2 600 m a.s.l. on Chogoria. *Otomys tropicalis* was recorded at all elevations on Sirimon, except 2 800 m a.s.l., but only occurred at higher elevations on Chogoria (2 600–3 400 m a.s.l.). *Sylvisorex mundus* were recorded at 2 400–2 800 m a.s.l. on Sirimon and at 2 600–2 800 m a.s.l. on Chogoria.

**Figure 2:**
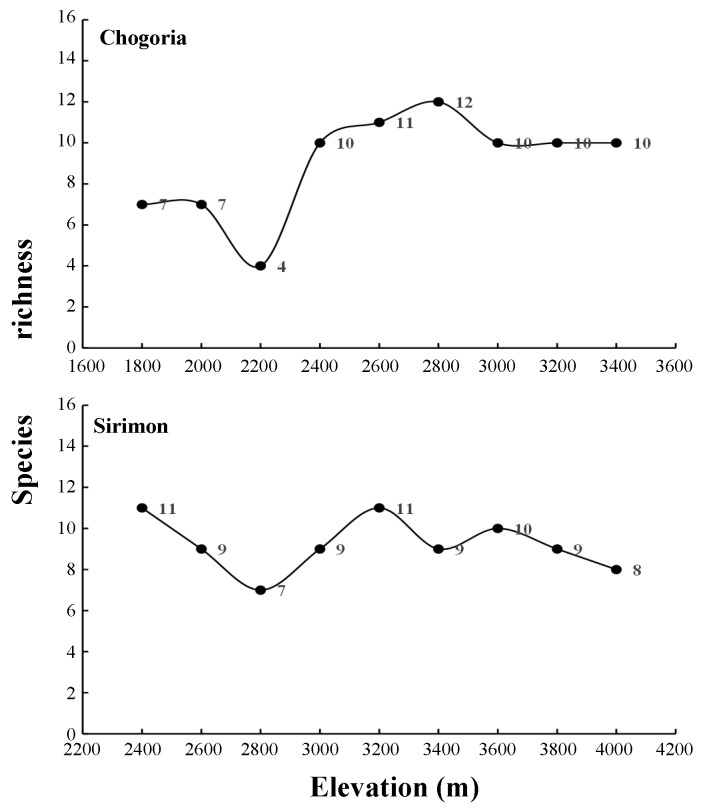
Distribution patterns of small mammal species richness with elevation on the Chogoria and Sirimon slopes of Mt. Kenya

The species richness and distribution patterns were different between Chogoria and Sirimon. On Chogoria, species richness was highest at mid-high elevations, with a peak at mid-elevation (2 800 m), whereas the lowest species richness was recorded at the lower elevation of 2 200 m a.s.l. (Figure 2). On Sirimon, species richness peaked at 1 800 m and 3 200 m a.s.l., with the lowest species richness found at 2 800 m a.s.l. Species richness on Chogoria was relatively even across the different elevations, with only a marginal decline along the sampled gradients (Figure 2).

### Small mammal abundance along elevation gradients

A total of 1 905 small mammal individuals were captured after 12 240 trap-nights, with a trap success rate of 15.5%. Eight hundred and ninety-eight (898) individuals were trapped on Chogoria and 1 007 on Sirimon. As expected, rodents were the most abundant along both transects: Chogoria (693) and Sirimon (823) (Table 3). Mean small mammal abundance for each elevation was 99.6±33.5 on Chogoria and 111.9±27.5 on Sirimon. The most abundant species was *Lophuromys zena* (27.7%), followed by *Hylomyscus endorobae* (15.5%), *Crocidura montis* (15.6%), and *Praomys jacksoni* (12%).

**Figure 3:**
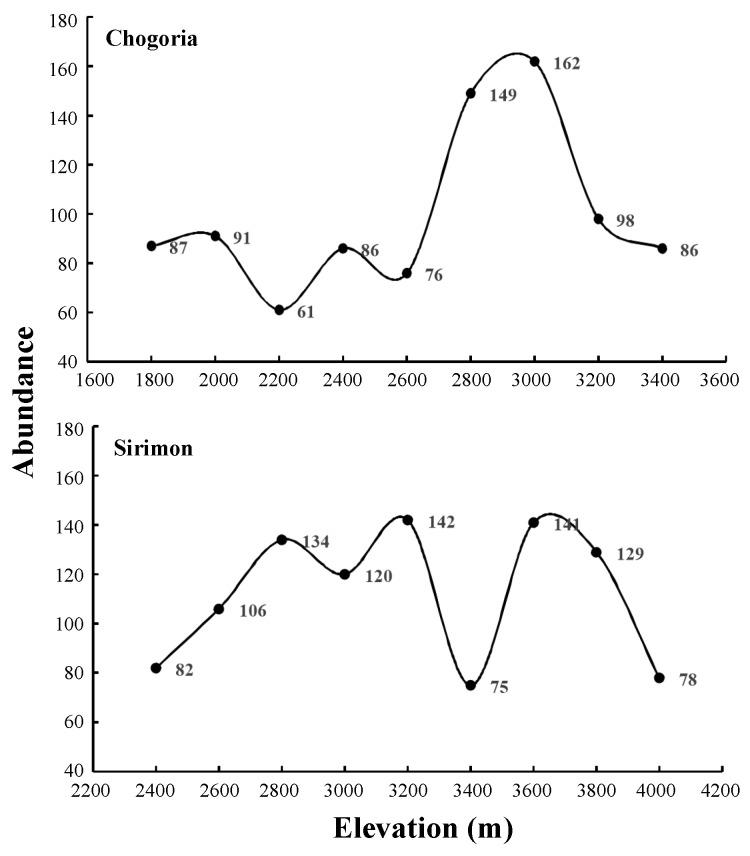
Distribution patterns of small mammal abundance with elevation on the Chogoria and Sirimon slopes of Mt. Kenya

The number of individuals captured per elevational transect varied from 62 to 162 on Chogoria and from 75 to 142 on Sirimon. On Chogoria, there were apparent peaks in small mammal abundance at 2 800 m and 3 000 m a.s.l. (Figure 3), where bamboo forest and *Juniperus-Hagenia* with large patches of tussock grassland were the main vegetation. The abundances in low elevation natural forest and high elevation moorland were relatively low. On Sirimon, abundance was higher at mid-elevations from 2 800–3 800 m a.s.l., except for an apparent dip at 3 400 m a.s.l.

### Medium- and large-sized mammals

Mt. Kenya is one of the largest forest conservation areas in Kenya. Although we focused on small mammals, we also conducted opportunistic recordings of medium- and large-sized mammals encountered both directly and indirectly. Most large mammals recorded, such as *Loxodonta africana* and *Syncerus caffer*, were difficult to observe directly because of thick forest cover, but we often encountered fresh tracks and scats of these species. Individuals of *Kobus ellipsiprymnus* were fairly easily seen in the open grasslands at lower elevations. *Cercopithecus mitis kolbi* and *Colobus guereza kikuyuensis* were very common in the forest canopies, especially on Chogoria. *Dendrohyrax arboreus* would call in the evening at lower elevations (1 800–3 000 m a.s.l.), especially in areas with continuous tree cover. The duiker (*Cephalophus nigrifrons*), hare (*Lepus victoriae*), spotted hyaena (*Crocuta crocuta*), and skunk (*Ictonyx striatus*) were rarely spotted. The giraffe (*Giraffa camelopardalis*) was only documented to occur on Chogoria by three teeth found near Lake Ellis at an elevation of 3 500 m (Mwebi et al., 2019).

## DISCUSSION

This study investigated the diversity and distribution patterns of small mammals on Mt. Kenya along different elevation gradients. We also compiled a checklist of mammals known to occur in the area. A total of 76 mammal species were recorded, including 46 small mammals and 30 large- or medium-sized mammals. We found higher small mammal species richness on Chogoria (24) compared to that on Sirimon (17). Chogoria had higher species diversity, probably because it occurs on the windward side of Mt. Kenya and receives more rainfall than the western leeward side (Sirimon). In addition, Chogoria had more diverse habitat types; six sampling transects between 1 800 m and 2 800 m a.s.l. occurred in forested habitats compared to four (2 400–3 000 m a.s.l.) on Sirimon. Thus, the larger forested habitat area and associated diversity of habitat niches on Chogoria may have contributed to the higher diversity of small mammals.

The compiled checklist of mammals is still incomplete as only a small part of Mt. Kenya’s ecosystem (271 000 ha) has been well surveyed. Most past surveys were only conducted for short periods over small areas and were generally opportunistic in nature. As such, a complete mammal list of the expansive and diverse habitats of the mountain has not yet been completed. Indeed, during the current field work, we trapped 25 species of small mammals, including at least one putative new species (*Mus* sp.). Importantly, we found that many taxa need revision. For example, individuals of the genus *Crocidura* were very difficult to identify using external morphology alone; hence, we used both molecular and morphometric characters to identify and classify these individuals. There were at least five species of *Crocidura* from the 345 individuals captured. However, most checklists of Mt. Kenya include only two species (*C. allex* and *C. olivieri*), except for Hollister (1919) who recognizes five. Similarly, two species of *Graphiurus* (*G. murinus* and *G. microtis saturates*) were recorded from Mt. Kenya by Hollister (1919). However, only *G. murinus* is widely accepted in recent publications (Young & Evans, 1993; Happold & Happold, 1989). Our Bayesian tree revealed two distinct clades in the genus *Graphiurus* with a genetic distance of 11.2%, suggesting an additional species, which may be *Graphiurus microtis saturates* recorded by Hollister (1919) or an undescribed species.

Other species of interest recorded in this survey included those of the genus *Tachyoryctes*; although we only trapped three individuals of this genus, many of their active and old nests were encountered in the grasslands, indicating that large numbers likely occur on Mt. Kenya. The taxonomy of *Tachyoryctes* is complex and is not well resolved in current work. For example, Musser & Carleton (2005) considers *T. ankoliae, T. annectens, T. audax, T. daemon, T. ibeanus, T. naivashae, T. rex, T. ruandae, T. ruddi, T. spalacinus and T. storey* as valid species, whereas Monadjem et al. (2015) confirms only *T. rex, T. annectens, T. ibeanus, T. spalacinus*, and *T. ruddi* as valid species from Kenya based on morphometric analysis and distinct biogeographical and ecological distributions of each species in the country. According to Monadjem et al. (2015), only two (*T. spalacinus* and *T. rex*) species of *Tachyoryctes* occur on Mt. Kenya. Thus, this taxonomic confusion of the genus *Tachyoryctes* in Kenya requires further research. In addition, although 104 bat species are known from Kenya (Musila et al., 2019), no bat survey has been undertaken on Mt. Kenya, and those listed in our supplementary table were recorded by opportunistic methods only. Furthermore, during a carnivore scat survey undertaken at the same time and on the same slopes as our survey, species of *Acomys* and *Thamnomys* were identified from animal remains collected on Mt. Kenya (Mwebi et al., 2019), which have not been reported previously on this mountain. Hence, it is expected that the species list of mammals from Mt. Kenya will increase in the future with additional surveys on bats, shrews, and rodents and as the taxonomic statuses of small mammals from this mountain become better understood.

As regions with high species abundance often occur at intermediate elevation bands (Bateman et al., 2010; Whittaker et al., 2001), the high abundance at mid-elevations on both the Chogoria and Sirimon slopes may be due to the interaction of temperature, water, and vegetation (Curran et al., 2012; Kok et al., 2012; Kryštufek et al., 2011; Linden et al., 2014; McCain, 2007). On Chogoria, small mammal abundance increased at 2 800–3 000 m a.s.l. in grassland and bamboo forest; whereas, on Sirimon, small mammals were most abundant in the tussock grassland (Table 1 and Table 3). The bamboo forest and tussock grassland had high habitat heterogeneity and complexity as well as water availability (e.g., streams). Interestingly, there was an apparent dip in abundance at 3 400 m a.s.l. on Sirimon. This anomalous decrease could be attributed to low habitat heterogeneity and high human disturbance (e.g., roads and settlements) due to the flatter terrain.

Our results revealed different species richness patterns between the two slopes of Mt. Kenya. On Chogoria, species richness was higher at high altitudes and peaked at mid-elevations (2 800 m a.s.l.). However, species richness showed little variation on Sirimon, which exhibited two peaks, one at mid-elevation (3 200 m a.s.l.) and one at lower elevation (2 600 m a.s.l.). These results are supported by previous studies that have shown that even different slopes of the same mountain habitat can possess distinct patterns of mammal distribution (Chen et al., 2017). Differences in climate and vegetation between slopes may also partly explain this phenomenon. Previous studies have revealed mostly hump-shaped distributions of small mammal species along elevation gradients, with richness peaking at mid-elevations (McCain, 2005, 2007). However, such elevational patterns of species richness between two opposing slopes of the same mountain ecosystem are, in general, rare. Interestingly, most previous studies on African mountains have not followed the hump-shaped distribution pattern (Kerbis et al., 1998; Kasangaki et al., 2003; Taylor et al., 2015). However, additional surveys are required to clarify these differences in elevational species richness patterns in African mountain ecosystems.

The Mt. Kenya ecosystem faces many conservation challenges, including annual fires, tree poaching, cultivation of the illegal but lucrative *Cannabis sativa*, and uncontrolled livestock grazing (Bennun & Njoroge, 1999). These habitat disturbances and fragmentation activities affect the distribution of both small and large mammals on Mt. Kenya. For instance, 21 individuals of *Surdisorex polulus* were recorded in the moorland and tussock grassland. Large areas of this habitat are razed at least once every two years, yet it is unclear how *Surdisorex polulus* individuals are affected by these fires. Furthermore, *Tragelaphus euryceros* has become rare on Mt. Kenya due to illegal bush-meat poaching, and it is likely that the abundance of this species may have declined dramatically over the years. Although Mt. Kenya is a large ecosystem with diverse habitats capable of sustaining a high diversity of mammals, biodiversity is increasingly threatened by human activities.

In conclusion, we examined small mammal diversity and distribution on the Chogoria and Sirimon slopes of Mt. Kenya and compiled a comprehensive checklist of mammals found on the mountain. Results demonstrated that there were more individuals of small mammal species on Sirimon than on Chogoria. Furthermore, although the highest abundance was observed at mid-elevations on both slopes, elevational species richness patterns differed between the slopes. The results of this study provide baseline information that can be used to monitor the effects of climate change on the distribution of mammal species in this ecosystem. In addition, to compile a comprehensive checklist of mammals of this mountain, more surveys, especially on bats, are needed.
